# Bio-inspired micropatterned thermochromic hydrogel for concurrent smart solar transmission and rapid visible-light stealth at all-working temperatures

**DOI:** 10.1038/s41377-024-01525-y

**Published:** 2024-08-21

**Authors:** Huaxu Liang, Xinping Zhang, Fuqiang Wang, Chunzhe Li, Weizhe Yuan, Weifeng Meng, Ziming Cheng, Yan Dong, Xuhang Shi, Yuying Yan, Hongliang Yi, Yong Shuai, Yi Long

**Affiliations:** 1 School of Energy Science and Engineering, Harbin Institute of Technology, 92 West Dazhi Street, Harbin, 150001 China; 2 School of Materials Science and Engineering, Nanyang Technological University, 50 Nanyang Avenue, Singapore, 639798 Singapore; 3 Faculty of Engineering, University of Nottingham, Nottingham, NG7 2RD UK; 4 Department of Environmental Engineering, Technical University of Denmark, Miljøvej 113, Kgs, Lyngby, 2800 Denmark; 5 Department of Electronic Engineering, the Chinese University of Hong Kong, New Territories, Hong Kong SAR, China

**Keywords:** Bioinspired materials, Micro-optics

## Abstract

Thermochromic hydrogels exhibit a smart capacity for regulating solar spectrum transmission, enabling automatically change their transmissivity in response to the ambient temperature change. This has great importance for energy conservation purposes. Military and civilian emergency thermochromic applications require rapid visible-light stealth (VLS); however, concurrent smart solar transmission and rapid VLS is yet to be realized. Inspired by squid-skin, we propose a micropatterned thermochromic hydrogel (MTH) to realize the concurrent control of smart solar transmittance and rapid VLS at all-working temperatures. The MTH possesses two optical regulation mechanisms: optical property regulation and optical scattering, controlled by temperature and pressure, respectively. The introduced surface micropattern strategy can arbitrarily switch between normal and diffuse transmission, and the VLS response time is within 1 s compared with previous ~180 s. The MTH also has a high solar-transmission regulation range of 61%. Further, the MTH preparation method is scalable and cost-effective. This novel regulation mechanism opens a new pathway towards applications with multifunctional optical requirements.

## Abstract (web summary)

Inspired by squid-skin, a micropatterned thermochromic hydrogel to realize the concurrent control of smart solar transmittance and rapid visible-light stealth at all-working temperatures is proposed.

## Graphical abstract

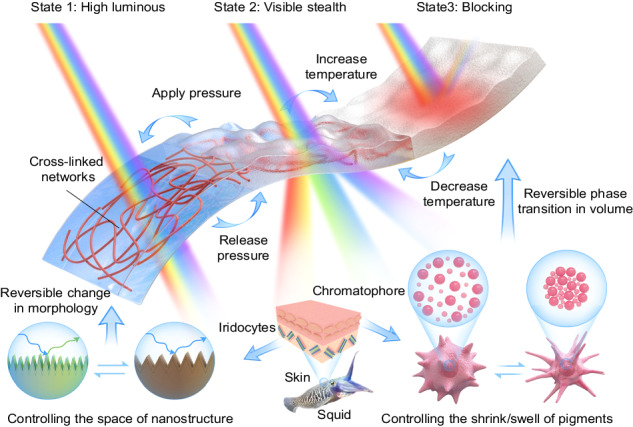

## Introduction

Large amounts of energy are consumed each year to offset the continuous solar heating and create a comfortable working environment, accounting for approximately 15% of global electricity consumption^1–3^. Thermochromic hydrogels exhibit a smart solar-spectrum transmission regulation that can be altered with temperature variation and have attracted tremendous interest in the fields of energy conservation^4^, dynamic memory devices^5^, and encryption^6^. For instance, the majority of cutting-edge thermochromic hydrogel is used for smart windows, which are designed to block solar transmission at high temperatures, contributing to cooling effects in summer. In contrast, it turns into transparent under low temperature, which can let more sunlight into the housing and thus heat up the building in winter. However, below lower critical solution temperature (LCST, 32 °C), the thermochromic hydrogel smart windows remain transparent, which disable the on demand visible-light stealth (VLS) functionality where privacy in building or sensitive information to be concealed are needed. Active heat source could be employed to address this issue, but the response times to enable such transition can be up to 180 s^7^, which could be an issue for both civilian and military applications that require additional rapid VLS activation. If VLS can be integrated into thermochromic hydrogel smart windows in military scenarios, rapid VLS could effectively improve camouflage and protect military equipment from detection; in civilian’s buildings, rapid VLS would realize both energy saving and privacy/information protection^8,9^; while in anti-counterfeiting area, Rapid VLS would offer dual anti-counterfeiting capabilities^10,11^.

The intrinsic characteristic of thermochromic hydrogels that provides smart solar transmission is a phase transition across the LCST^12–14^. Below LCST, polymer chains interact with surrounding water molecules, which give high transparency. Conversely, above LCST, the water molecules will be released from the polymer chains, which give low transparency. The physical mechanisms of a phase transition that lags significantly behind the temperature predetermine the ultraslow optical-regulation response to temperature variation^15–17^. Recently, studies have attempted to modify the structural and molecular properties of thermochromic hydrogels to accelerate the volume change rate^18–20^ and quicken the thermochromic response to temperature changes. These modifications include grafting side chains^21^, utilizing the elastic potential energy of the hydrogel network^22^, employing metal-phenolic complexes, DNA strands, micellar structures^23–26^, and more^27–29^. After structural and molecular modifications, the response times of thermochromic hydrogels, switching from high visible transparency to opacity, have been reduced from 180 to 84 s^30^; however, this is still far from the rapid response required for emergency information protection in military and civilian applications. Other studies have attempted to seek optical regulation strategies to realize VLS at all-working temperatures. A sodium acetate solution has been used to modulate visible light transmission under an active pressure stimulus^31^. However, this optical regulation strategy exhibits a VLS response time of 8 s and recovery time of ~600 s and cannot realize smart solar transmission.

Several squid species achieve smart camouflage with environmental coloration and rapid VLS within 1 s, which provides inspiration for the design of multifunctional regulation strategies. The distinctive tandem structures on squid skin utilize two different optical regulation strategies simultaneously: optical property alteration through internal material volume change and optical scattering control by surface microstructure variation^32^. The tandem structures on squid skin are composed of chromatophore pigment cells and iridocytes. The chromatophore pigment cells autonomously regulate the reversible change in the volume of interior pigments, and the iridocytes govern the reversible changes in morphology, thereby realizing surface microstructure control^33^.

Herein, inspired by the fast and concurrent optical regulation mechanisms of squid skin, we propose a novel micropatterned thermochromic hydrogel (MTH) to realize the concurrent control of smart solar transmission and rapid VLS at all-working temperatures. The proposed MTH simultaneously possesses two different optical regulation modes: internal-material optical property alteration and surface-layer optical scattering control, achieved using thermal and pressure stimuli, respectively. PNIPAm hydrogel is used as the MTH substrate to regulate solar transmission through reversible volume phase transitions under thermal stimulation (20–40 °C), endowing smart solar transmission (from 16% to 77% transmittance). A surface micropattern strategy was introduced for the MTH to arbitrarily switch between normal and diffuse solar transmission under pressure stimulation to realizing VLS within 1 s compared with the previous 180 s. Besides, the MTH can realize the VLS at all working temperature. Importantly, this newly designed MTH was prepared using a scalable and cost-effective approach, with the potential for large-scale application with multifunctional optical requirements, such as in smart windows, military installations, smart plant factories, and anti-counterfeiting.

## Results

### Design and optimization

**Fig. 1:**
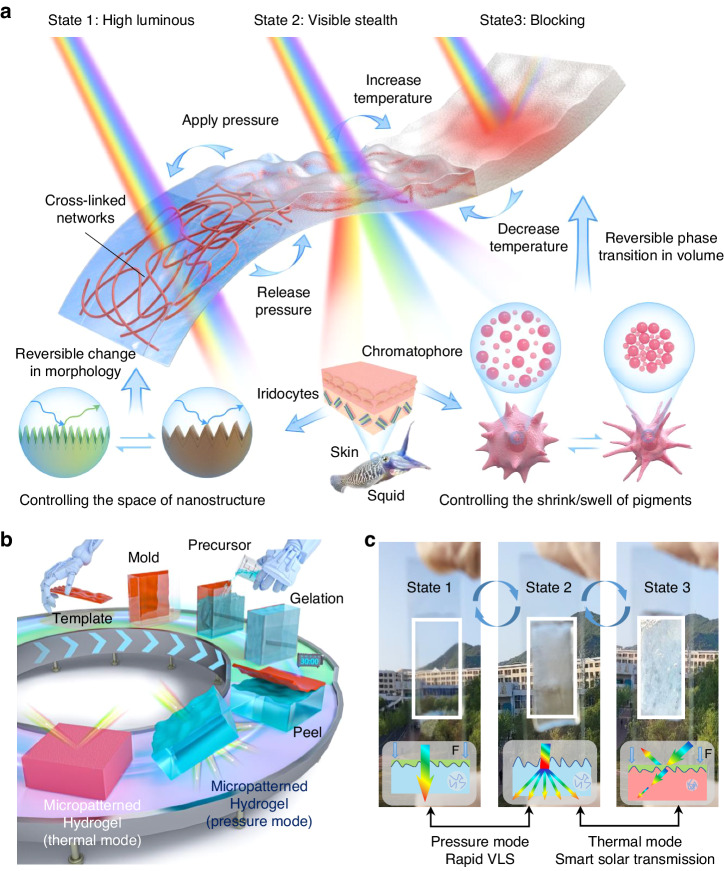
Schematic of the MTH. **a** Mechanisms of light transfer for the MTH under two-dimensional thermal and pressure stimuli, and of color changes in squid skin controlled by chromatophore and iridocytes cells; **b** proposed template-assisted fabrication of MTH; **c** states of MTH under thermal and pressure modes

The MTH comprises a PNIPAm hydrogel substrate and a micropatterned surface that mimics the tandem structure of squid skin (Fig. 1a). The PNIPAm hydrogel substrate regulates solar transmission through a reversible volume phase transition under thermal stimulation, enabling smart solar transmission and VLS at high temperatures. The micropatterned surface of the MTH is engineered to possess a surface microstructure with random roughness. Such surfaces can modulate and scatter light in various directions, rendering targets imperceptible^34^. Specifically, the introduced surface micropattern strategy for MTH can arbitrarily switch between normal and diffuse solar transmission through the change of surface morphology under a pressure stimulus, endowing rapid VLS at low temperatures.

The MTH was designed to be prepared using a scalable and cost-effective template-based method (Fig. 1b). A low-cost sandpaper was selected and attached to planar glass to serve as a random rough surface template. A mold equipped with the sandpaper template was filled with a precursor substance that subsequently underwent gelation. After gelation, the template was removed to form an MTH. This simple and scalable approach provides a robust strategy for fabricating MTH. The MTH was engineered to switch between three working states (Fig. 1c), allowing a single MTH unit to perform the concurrent regulation of smart solar transmission and rapid visible-light stealth on demand. In state 1, the pressure is applied, and the light can transmit directly through the MTH, so the MTH in state 1 exhibits high luminous. In state 2, the pressure is released, and the surface microstructure with random roughness of the MTH modulates and scatters light in various directions, rendering targets imperceptible. Therefore, the MTH in state 2 exhibits low luminous. In state 3, a volume phase transition of the MTH happens under thermal stimulation, resulting in high solar absorption and low reflection, so the MTH in state 3 can block sunlight under high temperature. The direction of the applied pressure is perpendicular to the surface of the MTH, (Supplementary Note 1, Fig. S1).

**Fig. 2:**
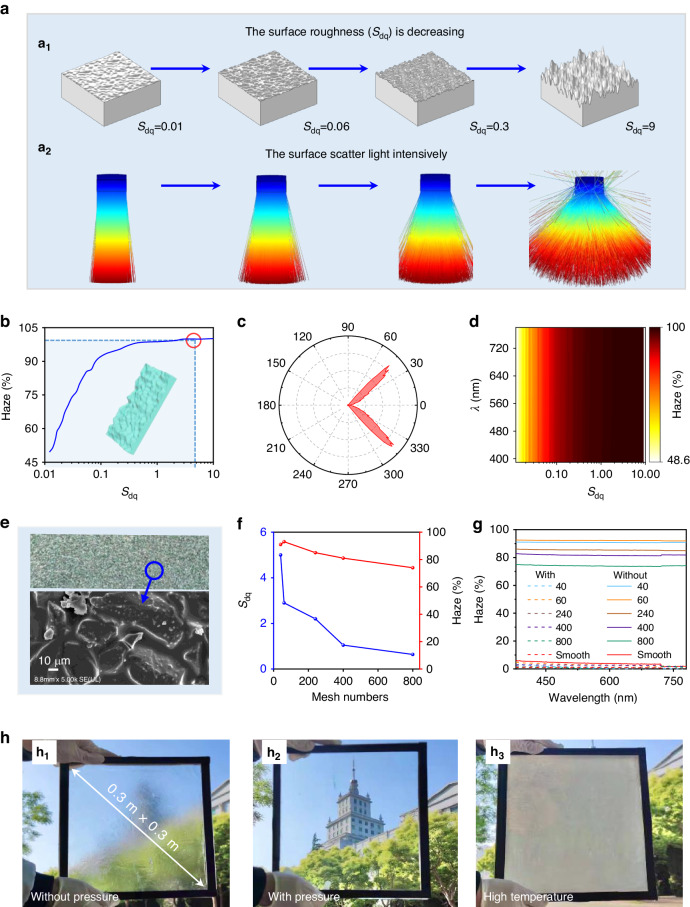
Design and optimization of MTH. **a** Light scattering behavior of Gaussian rough surfaces with different *S*_dq_; **b** effects of *S*_dq_ on the haze of Gaussian rough surfaces; (**c**) BTDF of the rough surfaces; **d** relationship between *S*_dq_, wavelength, and haze for rough surfaces; **e** SEM images of the sandpaper surface; **f** the corresponding *S*_dq_ and haze of MTH prepared by sandpaper templates with various mesh numbers; **g** the haze of MTH with and without pressure in the 380–780 nm band; and **h** different states of a large 30 cm × 30 cm MTH prepared with the scalable template-based process, H_1_: without pressure; H_2_: with pressure stimulation; H_3_: with thermal stimulation

The root means square gradient (*S*_dq_) is a quantitative measure of surface roughness that characterizes the variability in the height and spatial distribution of a random rough surface^35^. A Monte Carlo method was used to generate random rough surfaces (Supplementary Note 2). The haze parameter was used to evaluate the scattering capability^36^, as indicated in (Mathematical description). A three-dimensional optical transmission model for the MTH with varied *S*_dq_ was established, and the scattering capability was simulated (Fig. 2a). The analysis indicated that for a surface microstructure with random roughness, possessing a larger *S*_dq_ exhibited a greater capacity to scatter light. Haze increased with increasing *S*_dq_ (Fig. 2b). When the *S*_dq_ value reached 3.0, the haze attained its peak value and remained at a constant level with further increases in *S*_dq_. The bidirectional transmittance distribution function (BTDF) of the random surface microstructure with roughness was also calculated (Fig. 2c; Supplementary Note 3). The BTDF indicates that a surface microstructure with random roughness can scatter light at angles of 30°–45°. The correlation between haze, *S*_dq_, and wavelength was also investigated (Fig. 2D). Haze demonstrates a comparatively limited reliance on wavelength yet a notably substantial reliance on *S*_dq_. Consequently, the *S*_dq_ value of the micropatterned surface is expected to be controlled at 3.0 during the fabrication process.

The sandpaper template had a random rough surface, as evidenced by scanning electron microscopy (SEM) images (Fig. 2e). The surface roughness of the sandpaper was determined based on the nominal mesh number. A small mesh number corresponds to high surface roughness. Sandpapers with mesh numbers of 40, 60, 240, 400, and 800 were used as templates to fabricate various MTHs. The surface roughness of MTH was characterized using 3D confocal microscopy (LEXT OLS5000). As the sandpaper mesh number increased, the surface roughness of the MTH decreased (Fig. S2). Correlations between the sandpaper mesh number, MTH *S*_dq_, and haze were studied (Fig. 2f). The *S*_dq_ values of the five MTHs fabricated using the various sandpaper templates were 5, 2.9, 2.2, 1.05, and 0.64, respectively. The respective haze values were 91%, 93%, 85%, 81%, and 74%. The *S*_dq_ of the MTH fabricated using sandpaper with mesh number of 60 was 2.9, which is close to the optimal value of 3.0. The corresponding haze reached a peak value of 93%, which surpasses the haze observed in the MTHs fabricated using sandpaper with mesh numbers of 40, 240, 400, and 800. Under the pressure stimulus, the haze of the five MTHs decreased to below 10%. After the pressure was released, the haze returned to prestressed levels. The spectral hazes of the MTHs were also measured with and without pressure (Fig. 2g). For the entire visible wavelength band, the spectral haze of the MTH fabricated using the 60-mesh surpasses that observed in the MTHs fabricated using sandpapers with mesh numbers of 40, 240, 400, and 800. Consequently, 60-mesh sandpaper was the optimal candidate for the fabrication template.

A large MTH with dimensions of 30 cm × 30 cm was prepared using the scalable and low-cost template-based process with the 60-mesh sandpaper, which was subsequently encapsulated between two glass panels to create an MTH-based smart window (Fig. 2H). At low-working temperatures (approximately 20 °C), this device exhibited high opacity without pressure (Fig. 2H_1_) because the random rough micropatterned surface of the MTH scatters light in numerous directions. Under a pressure stimulus, the device exhibited high visible transparency (Fig. 2H_2_) because the micropatterned surface of the MTH switches from diffuse solar transmission to normal solar transmission through surface morphological changes while the total solar transmission remains almost unchanged. At high-working temperatures (approximately 40 °C), the MTH is opaque, and the total solar transmission was significantly reduced (Fig. 2H_3_) because the PNIPAm hydrogel substrate switches from normal transmittance to absorption and reflection through a volume phase transition under thermal stimulation.

### Optical performance characterization

**Fig. 3:**
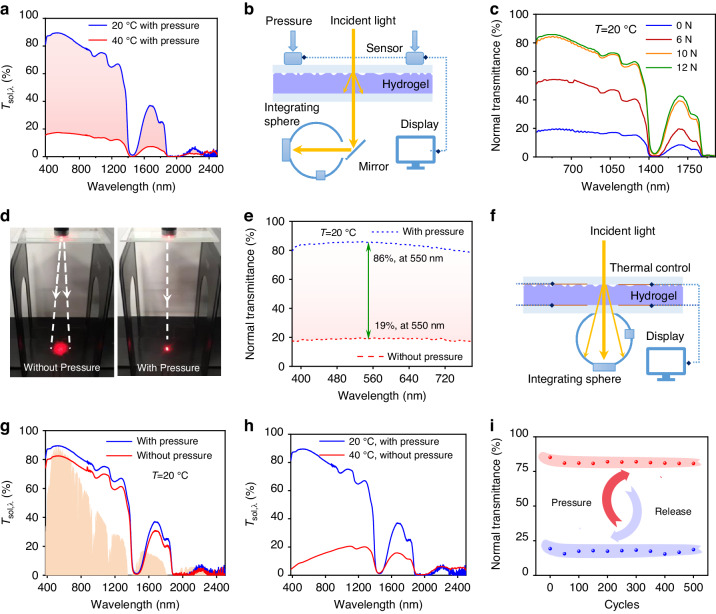
Optical characterization of MTH. **a** Solar transmittance *T*_*sol,λ*_ of the MTH, pre-pressed at 20 and 40 °C; **b** experimental setup for measuring transmittance *T*_*n,λ*_ under the pressure mode; **c** *T*_*n,λ*_ under the pressure mode; **d** scattering behavior of MTH; **e** visible spectrum transmittance *T*_vis,n,λ_ under the pressure mode, λ = 380–780 nm; **f** experimental setup for measuring *T*_sol,λ_ under the pressure mode; *T*_sol,λ_ under the pressure mode; **h** *T*_sol,λ_ under the superimposed modes of pressure and thermal stimuli; and (**I**) the stability and durability test

The hemispherical solar-spectrum transmittance (*T*_sol,λ_) of the MTH under pressure stimulation (pre-pressed) was measured at 20 and 40 °C (Fig. 3a). At low-working temperature (20 °C), the MTH has an ultra-high transmittance (*T*_sol_ = 77%). The mean integral of *T*_sol, λ_ is denoted as *T*_sol_, as expressed in the Mathematical description. Conversely, at high-working temperature (40 °C), *T*_sol_ decreased to only 16%, meaning the MTH has a solar transmission regulation capacity of 61%. In addition, the reflectivity of hydrogel at both high and low temperature were measured and displayed in Fig. S3. Both *R*_sol_ (reflectivity) of hydrogel at 40 °C and at 20 °C are 16% and 8%, respectively, and the *A*_sol_ (absorption) of hydrogel under 40 °C and under 20 °C are 68% and 15%, which suggests that the change in transmission is mainly due to absorption. The proposed MTH shows the converse optical property at low temperature and high temperature, and it demonstrates excellent performance in tuning *T*_sol,λ_ with temperature. The proposed MTH has a low *T*_sol,λ_ at high-working temperature helps MTH block solar radiation, which is beneficial for energy conservation.

The normal solar-spectrum transmittance (*T*_n, λ_) of the MTH with and without a pressure stimulus at low temperature (20 °C) was measured using a UV–VIS–NIR spectrophotometer retrofitted with precise pressure modules (Fig. 3b, Fig. S4). The pressure modules device has two force sensors, which is placed on the top of two ends of the MTH. The detail about measuring force is in the Supplementary Note 4. At low-working temperature (20 °C), the *T*_n, λ_ of the MTH increased with increasing pressure (Fig. 3c). *T*_n,λ_ reaches its peak value when the MTH is applied with 10 N, remaining unchanged with the further increases in pressure. When the MTH was beamed by a collimated red laser (610 nm) at a low working temperature, its micropatterned surface, without any pressure stimuli, scattered the red laser to a diffuse circular area. This diffusivity renders objects behind the MTH non-visible (Fig. 3d). After imposing pressure, the collimated red laser passed through the MTH with little scattering, rendering objects visible. A similar scattering phenomenon at low working temperature was observed when the MTH was beamed by collimated green light (532 nm) (Fig. S5). To exactly characterize VLS capability at low-working temperatures, the normal spectral transmittance of the MTH for the visible spectrum (*T*_vis,n,λ_) was measured (Fig. 3e). *T*_vis,n,λ_ was greater than 80% when pressure was applied to the MTH (visible state), and is greatly reduced to below 20% after the pressure is released (VLS state). Taking λ = 550 nm in the visible spectrum as an example^37^, Δ*T*_n,550 nm_ can reach 67% for the pressure variation mode. Considering the standard luminous efficiency function of photopic vision in the spectrum of 380–780 nm^38^, *T*_lum_ (as expressed in Mathematical description) was 86% at the visible state (20 °C, with pressure), while subsequent pressure release resulted in a decrease in *T*_lum_ from 86% to 19%, achieving a VLS state. The proposed MTH demonstrates that it can arbitrarily switch between normal and diffuse transmission through pressure, which can be used to tune the scattering in response to pressure change for rapid VLS and privacy protection. More importantly, the response time from highly visible to opaque was within 1 s, enabling a rapid VLS compared with the previous thermochromic response times of 180 and 84 s, as well as the previous pressure response time of 8 s and recovery time of approximately 600 s for a sodium acetate solution(See the Application Demonstrations section for details).

The principle of measuring the total solar transmittance (*T*_sol,λ_) of MTH with and without a pressure stimulus is illustrated in Fig. 3f. At low-working temperature (20 °C), the total solar transmittance of the MTH changed only slightly (10%) with and without pressure stimulus (Fig. 3g). By comparing Fig. 3g with Fig. 3c and e, it can be easily concluded that the pressure stimulus only switches the diffuse solar transmission to normal solar transmission while the total solar transmittance of the MTH remains almost unchanged.

The *T*_sol_ of MTH under the combined conditions of a low temperature (20 °C) and pressure stimulation was 77%, while at high temperature (40 °C) without any pressure stimulus it decreased to 13% (Fig. 3h). The normal transmittance of the MTH remained almost unchanged after 500 cycles of pressure application and release, demonstrating its high stability and durability (Fig. 3i). Moreover, the MTH exhibited enhanced performance in total solar transmittance under both thermal and pressure stimuli compared to most thermal-^39–41^, magnetic-^42^, hybrid-^43^, mechanical-^44,45^, hydro-^46,47^, and electric-^48,49^ based methods (see Fig. S6). For example, the thermal-based method employed PNIPAm hydrogel and showed excellent solar modulation of 58.4%, the magnetic-based method employed 1D Fe_3_O_4_@SiO_2_ nanochains and showed brilliant luminous-transmission regulation of 60%, and the mechanical-based method employed 3D nanocomposite film consisting of an ultrathin Al_2_O_3_ nanoshell and exhibited outstanding luminous-transmission regulation of 74%. It demonstrates competitive solar-transmission regulation range ($\varDelta {T}_{{\rm{sol}}}$) of 61% and excellent luminous-transmission regulation range ($\varDelta {T}_{{\rm{lum}}}$) of 67% compared with previous works. Its solar-transmission regulation is comparable to the thermal-based method and its luminous-transmission regulation is similar to the mechanical-based method. Meanwhile, both its solar-transmission regulation and luminous-transmission regulation have been significantly improved compared with the existing hybrid-based method. These results provide evidence that the MTH possesses a fast and concurrent optical control mechanism, whereby it exhibits simultaneous smart solar transmission regulation and rapid VLS capability through its response to both passive thermal and active pressure stimuli.

### Application demonstrations

**Fig. 4:**
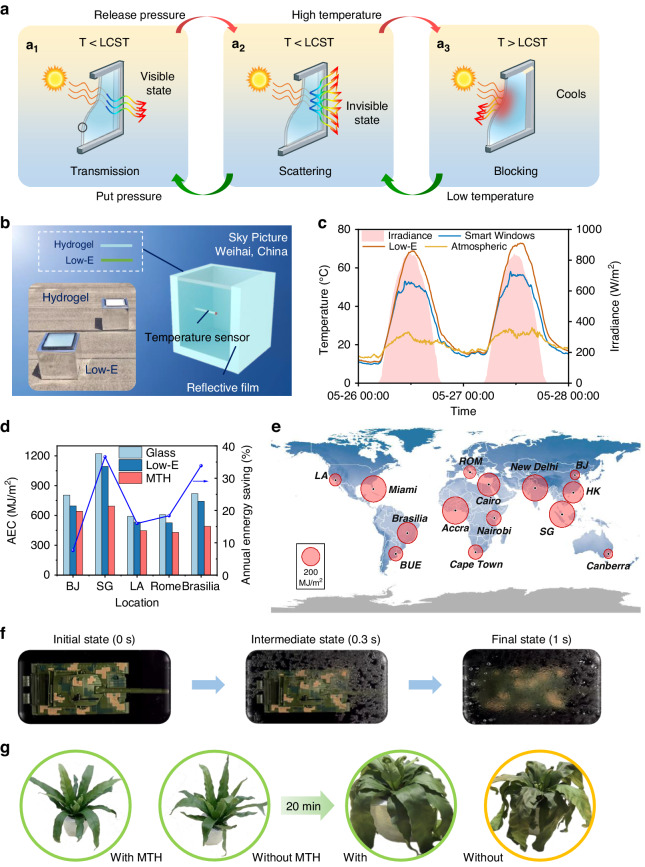
Application demonstration of MTH. (**a**) Working schematic of MTH-based smart windows; (**B**) setup for the thermal measurements of smart MTH-based and Low-E windowed houses in Weihai, China; (**c**) temperature of ambient and cavity air for the smart and Low-E windowed houses, and solar irradiance; (**d**) calculated AEC of the smart, Low-E, and glass windowed houses, and the energy-saving ratio of the smart window compared to the Low-E window in BJ, SG, LA, Rome and Brasilia; (**e**) the saved AEC for globally typical cities, the values are scaled to the area of the circles; (**f**) demonstration of MTH used for rapid visible stealth of a military tank; and (**g**) a demonstration of MTH protecting a plant from overheating

We present four viable application scenarios with multifunctional optical requirements for MTH. The first application involves employing MTH as a smart window to satisfy the dual demands of energy conservation and protecting privacy (Fig. 4a). The MTH-based smart window was fabricated by encapsulating MTHs between two glass panels. An MTH-based window can intelligently regulate the total solar transmission with variations in temperature, thus blocking strong sunlight from heating a room and yielding energy conservation benefits. Further, an MTH-based smart window can switch from normal to diffuse transmission when pressure is released, transforming from a transparent to an opaque state within 1 s and realizing rapid personal privacy protection. To evaluate the energy-saving efficiency of MTH-based smart windows, two model houses were fabricated. One house had an MTH-based smart window, while the other had Low-E window (Fig. 4b).

The sizes of both the smart and Low-E windows were 30 cm × 30 cm. The Low-E glass window used in this experiment is double-glazed, and the MTH window is also two layers of glass. During clear weather in Weihai city, China (37°30′45″N, 122°06′55″E), an outdoor energy-saving test was conducted. The air temperature inside the houses were measured continuously for 48 h. The specific outdoor measurement conditions were presented in Fig. S7, including the measurement instruments, the relative humidity, and wind speed corresponding to Fig. 4c. For the outdoor experiment, the relative humidity and wind speed were recorded by the atmospheric hygrometer (NH121, the precision was 0.1%) and anemometer (NHFSXY2808), as shown in the Fig. S7 A_1_ and A_2_. The solar irradiance was recorded by the solar radiometer (TBQ-2, the precision was <5%), as shown in the Fig. S7 A_3_. The temperature was measured using thermocouples (MT-K-08F, the precision was 0.01 °C). A multichannel temperature inspection device (SH-X, the precision was 0.1 °C) was used to obtain the real-time temperature, as shown in the Fig. S7 A_4_. The relative humidity and wind speed are shown in Fig. S7B. The Low-E glass window is set as the control experiment. The initial inside temperature of 41.5 °C swiftly rose to 70 °C within 3 hours (from 8:00 to 11:00) and reached a plateau at 72.5 °C (a 31 °C increase). In contrast, the phase transition of the MTH window resulted in a 15.9 °C increase after 3 hours. Furthermore, during office hours (9:00–17:00) when significant cooling was needed, the indoor air temperature in the MTH-based smart-windowed house consistently remained more than 8 °C lower than that in the Low-E-windowed house, while the indoor air temperatures were nearly identical at other times when cooling was unnecessary (Fig. 4C).

To further investigate the energy-saving potential of the MTH-based smart window, an energy consumption analysis of the model house was performed (Supplementary Note 5, Tables S1 and S2). The size of the house was 8 m × 8 m × 3 m, and four windows measuring 4 m × 2 m were installed in the center of the house (Fig. S8). The annual energy consumption (AEC) of a house with an MTH-based smart window was compared with that of a house with a common glass window and Low-E window in various locations, including Beijing, Singapore, Los Angeles, Rome, and Brasilia (Fig. 4d). The AEC of houses with the smart window is much lower than those with common glass or Low-E windows. Taking Singapore as an example, the AEC of a house with a smart window is 694 MJ/m^2^, whereas the AECs of houses with Low-E windows and common glass windows are 1094 and 1222 MJ/m^2^, respectively. Furthermore, the AECs of houses with MTH-based smart windows, common glass windows, and Low-E windows were calculated on a global scale (Supplementary Note 5, Fig. S9). A house with a smart window could save over 200 MJ/m^2^ per year compared with common glass and Low-E windows, in most cities (Fig. 4e, Fig. S10). Therefore, the utilization of MTH-based smart windows yields superior energy-saving performance compared to common glass windows and even Low-E windows.

The second application pertains to the utilization of MTH to attain a rapid VLS for a military tank model (Fig. 4f, Movie S1). When the MTH is subjected to a pressure stimulus, the military tank model is perceptible to the human eye. However, under emergency conditions such as enemy reconnaissance, a prompt release of pressure can instantaneously render the tank model invisible within 1 s to prevent detection by enemy visual systems. Therefore, the MTH can enable a rapid VLS since the response time from highly visible to opaque was within only 1 s, compared with the previous thermochromic response times of 180 s and 84 s^7,30^.

The third potential application of MTH is a smart plant factory (Fig. S11). The MTH can be used as a smart optical shield to protect plants from excessive solar radiation (Fig. 4g, Movie S2). In the absence of MTH (shielded by common glass), the plant leaves turned from green to yellow within 20 min of exposure to direct sunlight. Shielded by MTH, plant leaves maintained their green coloration. The employment of MTH demonstrates its ability to intelligently block solar radiation and effectively lower the ambient temperature surrounding the plant by approximately 6 °C compared to common glass (Fig. S12, Fig. S13).

The fourth application is in dual-level anti-counterfeiting. In its low-temperature state, an anti-counterfeiting MTH is barely transparent to the eye. However, when the anti-counterfeiting MTH was touched by hands for approximately 120 s, it changed from transparent to opaque owing to thermochromic effects, indicating that the anti-counterfeiting MTH had a temperature-sensitive response (Fig. S14, Movie S3). A pressure stimulus was subsequently applied to the anti-counterfeiting MTH to investigate the pressure response (Fig. S15, Movie S4). Words beneath the anti-counterfeiting MTH were revealed upon the application of pressure within 1 s. The pressure response exhibited by the anti-counterfeiting MTH facilitates the implementation of pressure encryption, thereby enhancing information safeguarding.

## Discussion

In this study, inspired by the fast and concurrent optical regulation mechanisms of squid skin, we proposed a novel micropatterned thermochromic hydrogel (MTH) to realize the concurrent control of smart solar transmission and rapid visible-light stealth (VLS) at all-working temperatures. The MTH possesses two different optical regulation modes: smart solar transmission and rapid VLS, controlled by temperature and pressure, respectively. This novel regulation mechanism opens a new pathway toward applications with multifunctional optical requirements.

The PNIPAm hydrogel substrate regulates solar-spectrum transmission through a reversible volume phase transition under thermal stimulation (20–40 °C), enabling a smart solar transmission (from 16% to 77% transmittance) and VLS at high temperature (40 °C). Specifically, the introduced surface micropattern strategy for MTH can arbitrarily switch between normal and diffuse transmission through reversible changes in morphology under a pressure stimulus and thus realize rapid VLS within 1 s, far faster than the current thermochromic hydrogel response times of 180 s. Besides, the MTH can realize the VLS at all working temperature. The practical usefulness of the proposed MTH was demonstrated in smart windows, effectively fulfilling the dual requirements of energy conservation and privacy protection.

MTH-based smart windows maintain indoor air temperature 8 °C lower than Low-E windows at office hours (9:00–17:00) when strong cooling is required, resulting in an annual energy saving of over 200 MJ/m^2^. In addition, the practical utility of the MTH was demonstrated for military equipment requiring rapid VLS. The MTH can also be utilized in smart plant factories and anti-counterfeiting. Importantly, the MTH is fabricated using a scalable, cost-effective, and bottom-up approach, which provides potential for large-scale applications. Future research efforts should focus on further optimizing the MTH and overcoming the identified challenges. For instance, customize the microstructure of MTH may further improve the optical regulation performance. Besides, the sandpaper template is disposable and cannot be reused, which will increase the cost for fabricating the MTH. This issue will worsen when fabricating larger quantities and larger-sized MTH to facilitate commercialization. Additionally, future research can also focus on exploring the broader application of MTH beyond windows, including its potential utilization on building roofs and walls.

## Materials and methods

### Raw materials

The N-isopropylacrylamide monomer (NIPAM, 98%, Mw = 113.16) was purchased from Macklin. The N,N′-methylenebis (acrylamide) (BIS, 99%, Mw = 154.17) crosslinker, potassium persulfate (KPS, 99.99%, Mw = 270.32) initiator, and N,N,N′,N′-tetramethylethylenediamine (TEMED, 99%, Mw = 116.2) were purchased from Sigma-Aldrich. All the materials were used without further purification. Deionized water was used in all experiments.

### Preparation of MTH

First, 0.535 g of NIPAM, 0.0125 g of BIS, and 0.025 g of KPS were dissolved in 10 mL of deionized water in a beaker and mixed using a magnetic stirring apparatus until completely dissolved. The mixed solution was then bubbled with N_2_ gas for 15 min to eliminate dissolved oxygen. Next, the mixed solution (as a precursor substance) was transferred to a glass mold attached to low-cost sandpaper. Fifty microliters of TEMED (catalyst) were added to the glass mold, and the container was sealed immediately. The solution was left to gelate. Once the gelation process was complete, the template was removed to form an MTH. All preparation processes were carried out at room temperature.

The MTH-based smart window was fabricated through the addition of 250 mL of the aforementioned mixture to a hollow and double-glazed glass panel with a sandpaper template. After gelation, the template was removed, and the MTH was washed in the deionized water to remove the residual precursor. Then, take the MTH out from the deionized water without any further treatment, and MTH was encapsulated by two layers of glass. The corresponding dosages of the materials were 13.375 g NIPAM, 0.3125 g BIS, 0.625 g KPS, and 250 mL of deionized water.

### Characterization

The transmittance spectra of the MTH were measured using an ultraviolet-visible-near-infrared (UV-VIS-NIR) spectrophotometer (SolidSpec-3700; Shimadzu, Japan). Before the measurement, we used air and a standard whiteboard to calibrate the transmittance and reflectivity of the instrument. During the measurement, all the MTH samples were measured under the same ambient temperature (20 °C). And the transmittance and reflectivity of MTH were measured three times. As for temperature measurement, we measured the same temperature using multiple thermocouples at the same time. As for pressure measurement, high-precision pressure sensor (ZNHM-I, Zhongnuo, China, the sensitivity was 1.0 mV/V) was employed in this study.

#### Outdoor experiment

The cooling performance of the MTH-based smart window was measured on a building roof in Weihai, China (37°31′46″N, 122°4′40′′E). The rooftop measurement system consisted of two polyfoam chambers wrapped in reflective films, temperature sensors, and glass samples. Low-E glass and smart windows, both 30 cm × 30 cm in size, were employed for the rooftop measurements. The size of the polyfoam chamber was 37 cm × 37 cm × 38.5 cm, and the size of the inside cavity was 28 cm × 28 cm × 29.5 cm. The temperature was measured using thermocouples (MT-K-08F, Lianyi, China, the precision was 0.01 °C). A multichannel temperature inspection device (SH-X, Lianyi, China, the precision was 0.1 °C) was used to obtain the real-time temperature. The asplenium nidus was employed to demonstrate the application of MTH in smart plant factory. In the experiment, the plants were placed in two transparent boxes made of acrylic sheets (the size was 30 cm × 30 cm × 30 cm), one of which was covered with MTH. Infrared images of the MTH were captured using a thermal infrared imager (Ti400, Fluke, America).

### Mathematical description

To assess the privacy mode performance under pressure mode, the *T*_lum_ is calculated by:

1 $${T}_{{\rm{lum}}}=\frac{{\int }_{380{\rm{nm}}}^{780{\rm{nm}}}{\varphi }_{{\rm{lum}}}(\lambda )\cdot {T}_{{\rm{vis}},{\rm{n}},\lambda }d\lambda }{{\int }_{380{\rm{nm}}}^{780{\rm{nm}}}{\varphi }_{{\rm{lum}}}(\lambda )d\lambda }$$

where *T*_vis,n,*λ*_ denotes spectral transmittance at normal direction, *φ*_lum_ (*λ*) is the standard luminous efficiency function of photopic vision in the wavelength range of 380–780 nm.

To assess the energy-saving mode performance under thermal mode, the *T*_sol_ is calculated by:

2 $${T}_{{\rm{sol}}}=\frac{{\int }_{300{\rm{nm}}}^{{\rm{2500nm}}}{I}_{{\rm{sol}}}(\lambda )\cdot {T}_{{\rm{sol}},\lambda }d\lambda }{{\int }_{300{\rm{nm}}}^{{\rm{2500nm}}}{I}_{{\rm{sol}}}(\lambda )d\lambda }$$

where *T*_sol,*λ*_ denotes spectral transmittance at hemispherical direction, *I*_sol_(*λ*) is the solar irradiance spectrum in the range of 300-2500 nm. And the $\varDelta {T}_{{\rm{sol}}}$ can be calculated by Eq. S15.

The light scattering behavior of random rough surface can be characterized by haze, which represents the percentage of the transmitted light that deviates from the incident light angle more than 2.5° to the total transmitted light, which can be expressed as:

3 $$Haze=\frac{{G}_{t,{\theta }_{i} > {2.5}^{\circ }}}{{G}_{t,{\rm{total}}}}\times 100 \%$$

where ${G}_{t,{\theta }_{i} > {2.5}^{\circ }}$ represent the flux of light that deviate from the angle of incident light by more than 2.5° and ${G}_{t,{\rm{total}}}$ represent the flux of total light.

### Supplementary information

Supplementary Materials for Bio-inspired micropatterned thermochromic hydrogel for concurrent smart solar transmission and rapid visible-light stealth at all-working temperatures

Movie S1

Movie S2

Movie S3

Movie S4

## Data Availability

All data are available in the main text or the supplementary materials.
