# Supplementary material for: Bio-inspired micropatterned thermochromic hydrogel for concurrent smart solar transmission and rapid visible-light stealth at all-working temperatures
Source: Light Sci Appl. 2024 Aug 21;13:202. doi: 10.1038/s41377-024-01525-y (PMC11339365; doi:10.1038/s41377-024-01525-y)
Supplement: Supplementary file 1 — Supplementary Materials for Bio-inspired micropatterned thermochromic hydrogel for concurrent smart solar transmission and rapid visible-light stealth at all-working temperatures [file 41377_2024_1525_MOESM1_ESM.pdf]

Supplementary Materials for

Bio-inspired micropatterned thermochromic hydrogel for concurrent smart solar transmission and rapid visible-light stealth at all-working temperatures

Huaxu Liang *et al.*

*Corresponding author: Fuqiang Wang; Yi Long.

Email: Wangfuqiang@hitwh.edu.cn; yilong@cuhk.edu.hk

This PDF file includes:

Supplementary Text
Figs. S1 to S16
Tables S1 to S2

Other Supplementary Materials for this manuscript include the following:

Movies S1 to S4

Supplementary Text

Note S1 The direction and application point of the applied pressure.

As illustrated in **Fig. S1**, the direction and application point of applied pressure are displayed. The direction of the applied pressure is perpendicular to the surface of the MTH, and the application point is on two ends of the MTH. The pressure is exerted through mechanical device.

Note S2 Light scattering behavior of rough surfaces with Gaussian statics

In order to model and simulate the light scattering behavior, rough surfaces with Gaussian statics can be generated using a Monte Carlo method outlined by Garcia and Stoll [50]. The principle of the Monte Carlo method is that the white noise random number is first transformed by fast Fourier transform, then the Gaussian rough surface power spectrum density is used to filter it in the frequency domain, and finally the inverse fast Fourier transform is used to obtain the 3D height data of the rough surface.

The specific steps of generating 3D random rough surface are as follows:

(1) Select the horizontal and vertical lengths of desired random rough surfaces as L_x and L_y. This region should contain enough peaks and troughs, and the sampling interval lengths (Δx and Δy) should satisfy the sampling theorem to ensure the accuracy of generating surfaces. Select the power spectral density function as $W(\boldsymbol{K})$. Where L_x=L_y=5000 μm, Δx=Δy=20 μm.

(2) Generate an independent Gauss random number matrix $\gamma_{m,n}$ (m=0~2M, n=0~2N) with mean μ and variance σ^2, where $M=\frac{L_x}{2\Delta x}$, $N=\frac{L_y}{2\Delta y}$, μ=0, and σ=1.

(3) Generation of complex random numbers as follows：

$$\hat{\gamma}_{mn}=\gamma_{mn}+i\beta_{mn}(m=1\sim 2M, n=1\sim 2N) \quad \text{(Eq. S1)}$$

(4) Calculation of the expansion coefficient $\hat{\eta}_{mn}$:

$$\chi_{mn}=W\left(\frac{2\pi m}{\boldsymbol{L}},\frac{2\pi n}{\boldsymbol{L}}\right),(m=0\sim M, n=0\sim N) \quad \text{(Eq. S2)}$$

$$\hat{\eta}_{mn}=\pi\sqrt{\frac{\chi_{mn}}{L_x L_y}}\hat{\gamma}_{mn} \quad \text{(Eq. S3)}$$

(5) Calculation of FFT:

$$\hat{h}_{pq}=\sum_{m=0}^{2M}\sum_{n=0}^{2N}\hat{\eta}_{mn}\exp(i(\frac{2\pi}{M}mp+\frac{2\pi}{N}nq)),(p=0\sim 2M,q=0\sim 2N) \quad \text{(Eq. S4)}$$

(6) Calculation of the surface height of the sample

$$h_{pq}=2\,\mathrm{Re}\left\{\hat{h}_{pq}\right\},(p=0\sim 2M,q=0\sim 2N) \quad \text{(Eq. S5)}$$

Random rough surfaces with different random characteristics can be obtained by substituting different power spectral density functions:

$$W(K_x,K_y)=\frac{S_qL_xL_y}{4\pi}\exp(-\frac{K_x^2L_x^2+K_y^2L_y^2}{4}) \quad \text{(Eq. S6)}$$

The geometric characteristics of random rough surface can be described by root mean square height (S_{q}), root mean square slope (S_{dq}) and correlation length (l).

The S_{q} can describe the average undulation height of random rough surface, which can be expressed as:

$$S_{\mathrm{q}}=\sqrt{\frac{1}{A}\iint_A z^2(x,y)dxdy} \quad \text{(Eq. S7)}$$

The S_{dq} can describe the average slope of random rough surface, which can be expressed as:

$$S_{\mathrm{dq}}=\sqrt{\frac{1}{A}\iint_A\left[\left(\frac{\partial z(x,y)}{\partial x}\right)^2+\left(\frac{\partial z(x,y)}{\partial y}\right)^2\right]dxdy} \quad \text{(Eq. S8)}$$

The l can describe the density of random fluctuations of height.

The spatial distribution of surface roughness is usually interrelated, and the surface autocorrelation function ($F(R)$) can describe this correlation property, which can be expressed as

$$F(R)=<z(r)z(r+R)> \quad \text{(Eq. S9)}$$

The distance when the normalized correlation coefficient(ρ) is reduced to 1/e is defined as the correlation length. The normalized correlation coefficient(ρ) can be expressed as:

$$\rho(R)=\frac{<z(r)z(r+R)>}{S_{\mathrm{q}}^2} \quad \text{(Eq. S10)}$$

When the S_{q} and incident zenith angle (θ_{i}) of rough surface and wavelength (λ) satisfies the relation as S-1, the geometric optical approximation can ensure the simulation accuracy of light scattering characteristics [51].

$$S_q \cos\theta_i / \lambda > 0.17 \qquad \text{(Eq. S11)}$$

Light scattering characteristics can be calculated using the geometric optics module in COMSOL Multiphysics@ software [52]. This module uses ray tracing method to model the propagation of electromagnetic waves, where the propagation of waves is treated as rays and can be reflected, refracted or absorbed. The refractive index of the hydrogel varies between 1.26-1.35 with the change in wavelength (**Fig. S16)** [17].

Note S3 Bidirectional transmitted distribution function

The spatial distribution of transmitted light scattering on material surface is characterized by bidirectional transmission distribution function (BTDF) [53]. Bidirectional transmission distribution function is defined as the ratio of transmitted radiation intensity and incident irradiance at a given zenith angle and azimuth angle, which can be expressed as:

$$BTDF(\theta_i,\varphi_i,\theta_t,\varphi_t)=\frac{dL_t(\theta_i,\varphi_i,\theta_t,\varphi_t)}{dE_i(\theta_i,\varphi_i)}=\frac{dL_t(\theta_i,\varphi_i,\theta_t,\varphi_t)}{L_i(\theta_i,\varphi_i)\cos\theta_i d\Omega_i} \qquad \text{(Eq. S12)}$$

where θ_i,φ_i represent the zenith angle and azimuth angle of incident light, θ_t,φ_t represent the zenith angle and azimuth angle of transmitted light, L_i,L_t represent the radiation intensity of incident and transmitted light, E_i represent the incident irradiance, Ω_i represent the solid angle of incident light.

Note. S4 The detail about measuring force.

The pressure modules device has two force sensors, which is placed on the top of two ends of the MTH. The force is exerted on two force sensors, applying force on the MTH through the force sensors. For each sensor, the area contacting the MTH is 3.14 cm^2. Therefore, the total value of applied force is the sum of the forces detected by each sensor. In the practice on windows in houses, the commercial electric linear actuator and mechanical device could be used to induce pressure.

Note. S5 Energy consumption analysis

To further investigate the energy-saving potential of this smart window, Energy Plus is used to establish a single-story house model and the "ideal-loads-air-systems" served with district

cooling and heating sources is used to simulate the annual energy consumption. The results represent the direct effects of different window types (MTH, low-e, common glass) on the heating and cooling load of the building. Specifically, the building dimensions are 8 m (length) × 8 m (width) × 3 m (height), the details of the building model are displayed in **Table S1**, and the insulation material meets the requirements of ASHRAE Standard 90.1. In this model, the total glass area of the building is 32 m^2, and the window wall ratio is 33.33%, evenly distributed on all four walls. The optical parameters of low-e and common glass used in the simulation were obtained from Ref. [38], and the optical parameters of MTH were obtained from experimental measurements, as shown in **Table S2**.

It is worth noting that the thermochromic performance of micropatterned hydrogels based smart window is achieved through the Energy Plus built-in thermochromic function. In the simulation, the interior temperatures are designed 18 ℃ in winter and 26 ℃ in summer. In winter, if the temperature is lower than 18 ℃, the building will be heated. In summer, if the temperature is higher than 26 ℃, the building will be cooled. During the operation of EnergyPlus, we set the MTH parameters under different temperature conditions, at the beginning of a specific time step calculation, the MTH glass temperature of the previous time step is used to find the closest temperature condition, and the corresponding MTH parameter will be used for the current time step calculation. We set the time step of EnergyPlus calculation as 10 min to reduce the deviation of calculation results caused by the lag of this iterative method. We selected cities in different regions of the world to predict the energy-saving performance (**Fig. 4E**): Los Angeles, CA, U.S.; Miami, FL, U.S.; Brasilia, Brazil; Buenos Aires, Argentina; Accra, Ghana; Rome, Italy; Cape Town, South Africa; Cairo, Egypt; Nairobi, Kenya; New Delhi, India; Singapore, Singapore; Hong Kong, China; Beijing, China; Canberra, Australia. The weather data adopted in this investigation are from the online resource provided by the U.S. Department of Energy (DOE) [54]

Note S6 Equivalent conductivity of the MTH window

The conductivity of the MTH window is set as 0.9 $W\cdot(m\cdot K)^{-1}$, which is an equivalent thermal conductivity of the glass/hydrogel/glass sandwich. Commonly, the conductivity of hydrogel is about 0.4~0.6 $W\cdot(m\cdot K)^{-1}$ [55], and the conductivity of building glass is about 0.9~1.3 $W\cdot(m\cdot K)^{-1}$ [56]. According to the equivalent thermal conductivity equation

$$\frac{d_1}{h_1}+\frac{d_2}{h_2}+\frac{d_3}{h_3}=\frac{d_1+d_2+d_3}{h_{equ}} \quad \text{(Eq. S13)}$$

the equivalent conductivity of the MTH window is calculated as 0.9 W·(m·K)$^{-1}$, where d_1, d_2 and d_3 are the thickness of glass, hydrogel and glass, respectively; h_1, h_2, and h_3 are the conductivity of glass, hydrogel and glass, respectively.

Note. S7 Additional mathematical description.

The annual energy consumption (AEC) of a house can be divided to two parts as follows:

$$\mathrm{AEC}=\mathrm{HEC}+\mathrm{CEC} \quad \text{(Eq. S14)}$$

where HEC is the heating energy consumption, and CEC is the cooling energy consumption.

The ΔT_{sol} can be calculated by

$$\Delta T_{sol}=T_{sol,40\,℃}\text{-}T_{sol,20\,℃} \quad \text{(Eq. S15)}$$

where $T_{sol,40\,°C}$ and $T_{sol,20\,°C}$ are integrated solar transmittance at 40 °C and 20 °C.

The relationship between absorption, transmittance, and reflection is as follows:

$$A+T+R=1 \quad \text{(Eq. S16)}$$

Fig. S1.

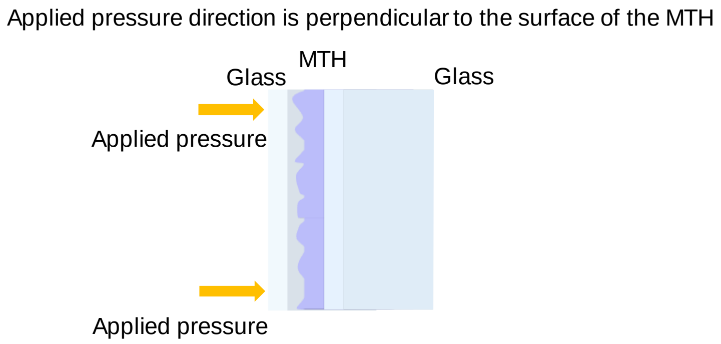

Fig. S1 The direction and application point of the applied pressure

Fig. S2.

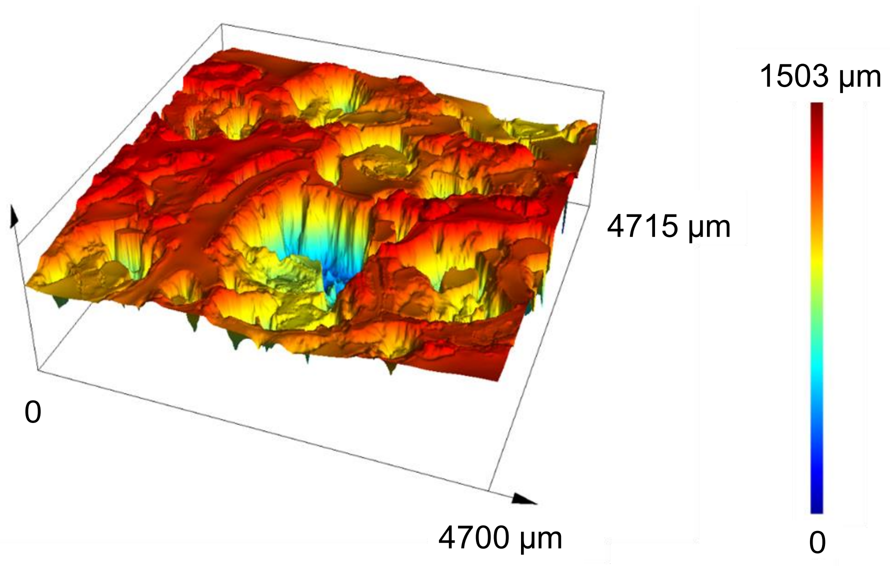

(a) 40 mesh number

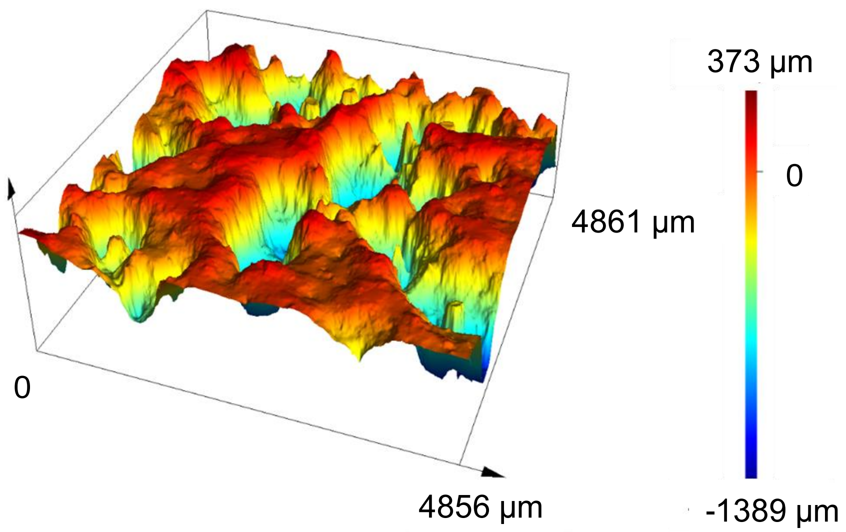

(b) 60 mesh number

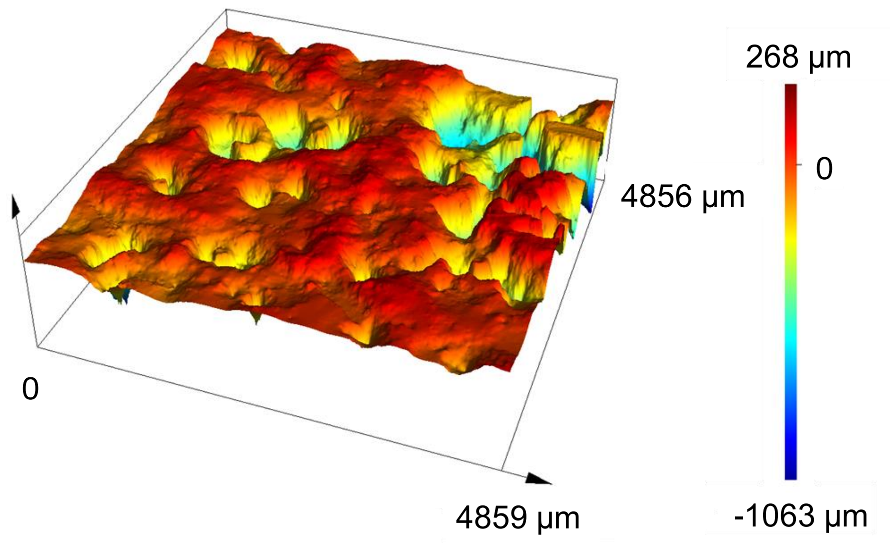

(c) 240 mesh number

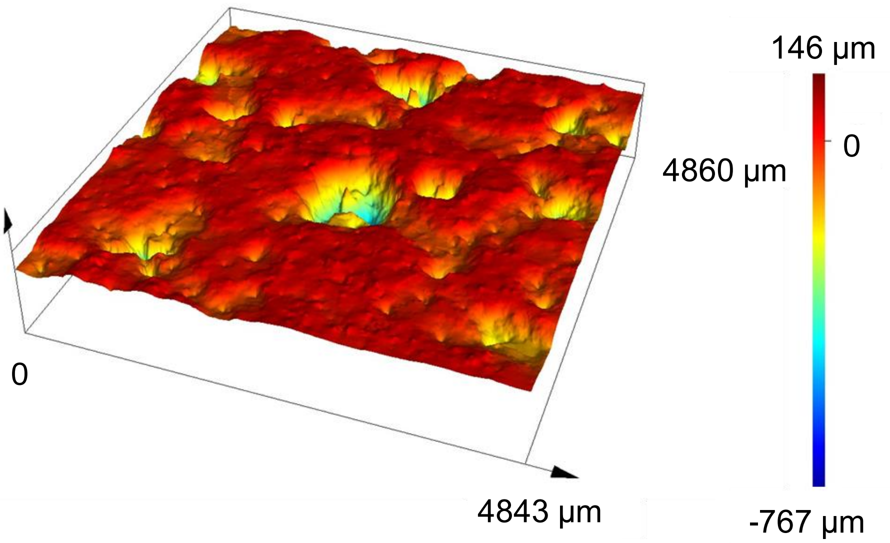

(d) 400 mesh number

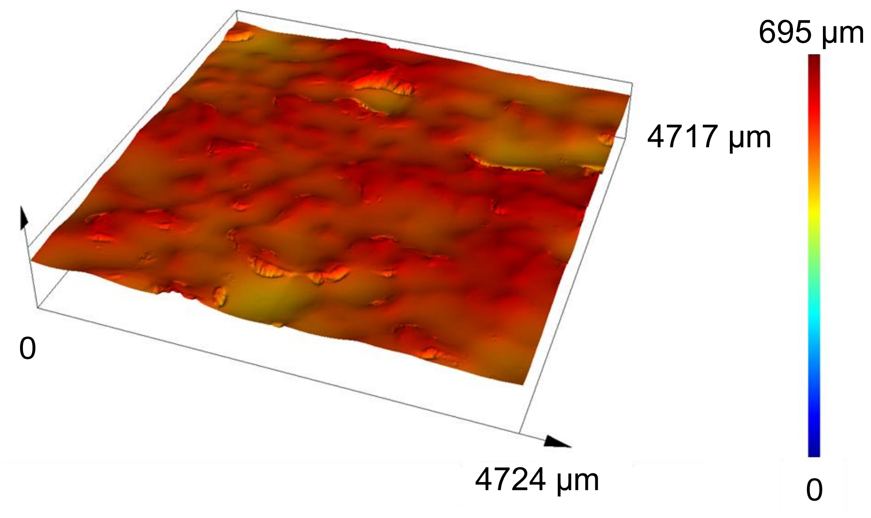

(e) 800 mesh number

Fig. S2. Roughness of MTH fabricated by different sandpaper (40, 60, 240, 400 and 800 meshes number)

Fig. S3.

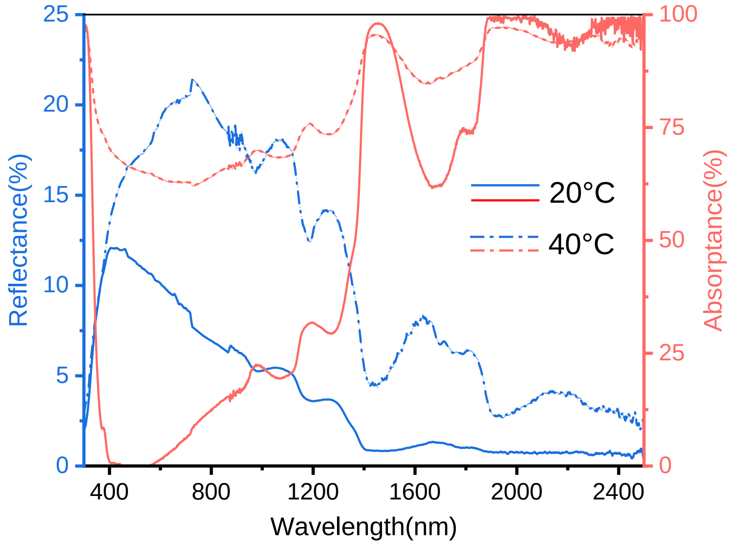

Fig. S3 The reflectivity and absorptivity of hydrogel under high temperature (40 °C) and low temperature (20 °C)

Fig. S4.

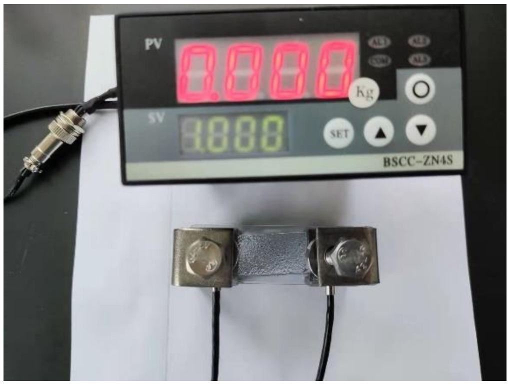

(a) the actual device for controlling pressure, which includes a mechanical actuator device for putting/release the pressure and two force sensors for testing the pressures

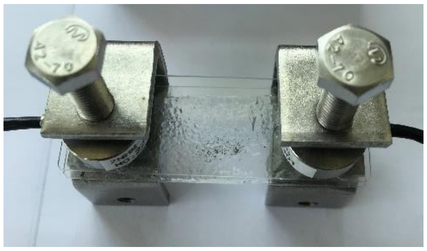

(b) mechanical actuator release pressure

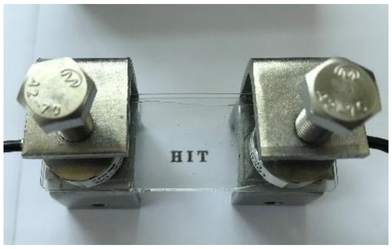

(c) mechanical actuator put pressure

Fig. S4 the device for applying pressure.

Fig. S5.

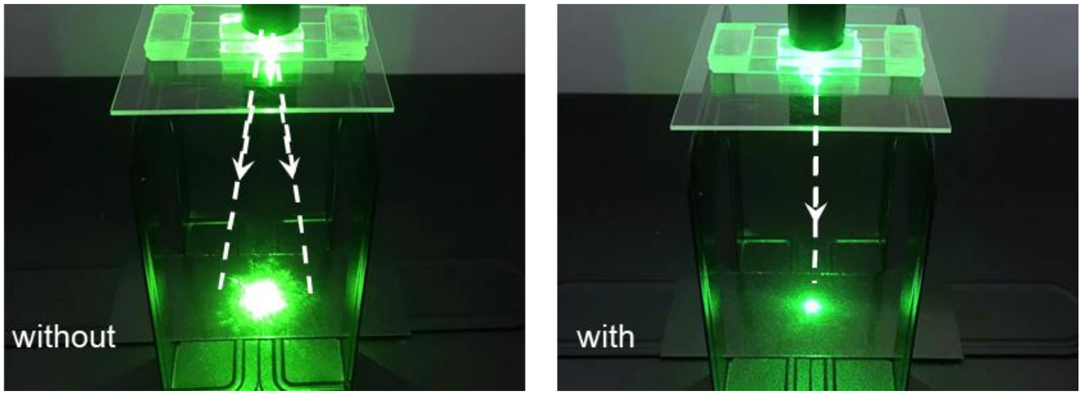

Fig.S5 the green light scattering behavior of MTH

Fig. S6.

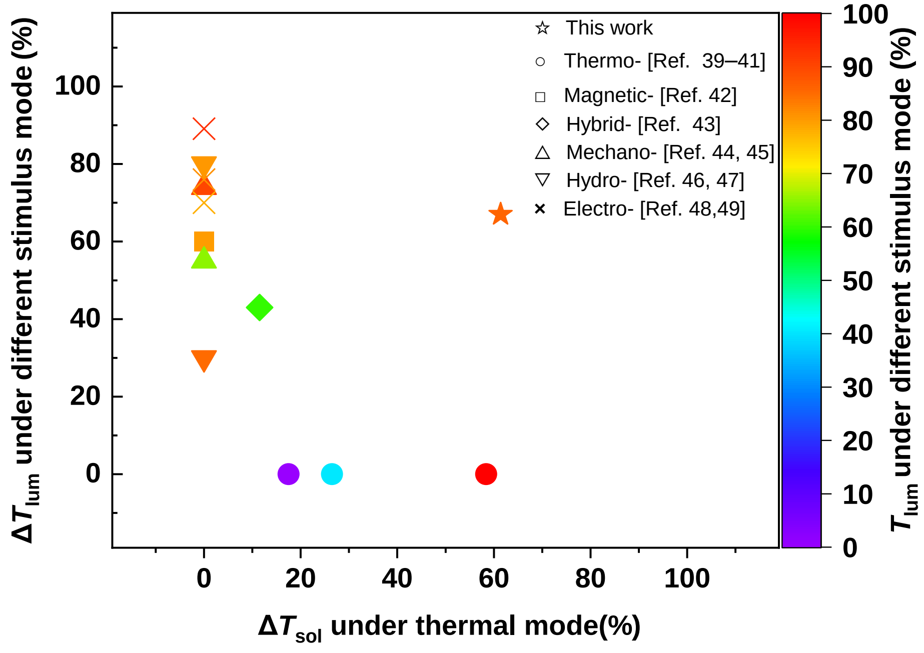

Fig.S6 The T_{lum} under pressure mode, ΔT_{lum} under pressure mode and ΔT_{sol} under thermal mode of this study and thermos-, magnetic-, hybrid, mechano-, hydro and electro-based methods.

Fig. S7.

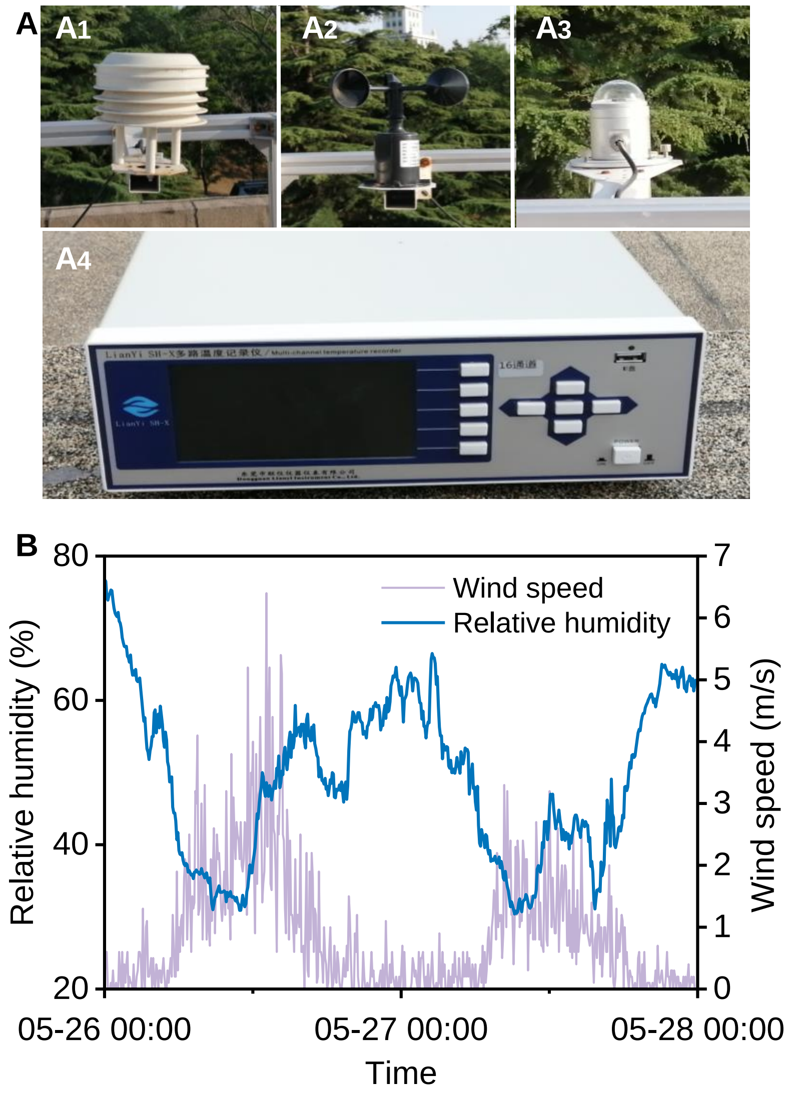

Fig.S7 (A) the outdoor measurement instruments, including (A_1) atmospheric hygrometer, (A_2) anemometer, (A_3) solar radiometer, and (A_4) temperature inspection instrument; (B) the relative humidity and wind speed

Fig. S8.

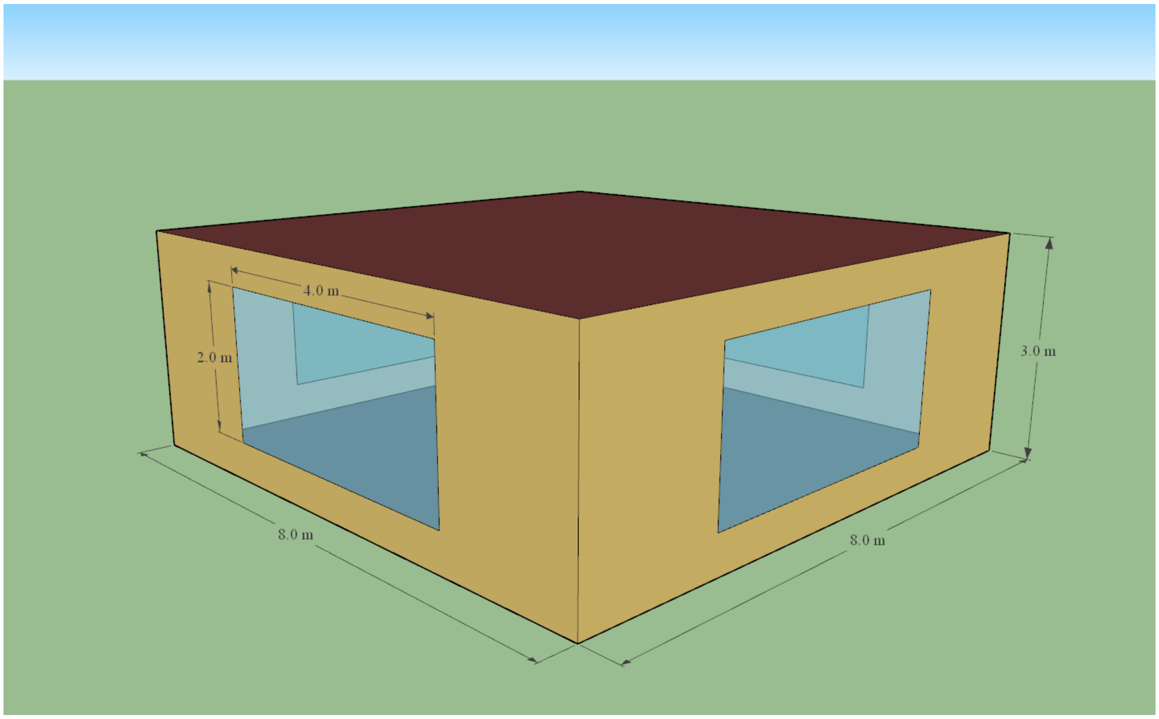

Fig.S8 the model of house with four windows in the center of wall.

Fig. S9.

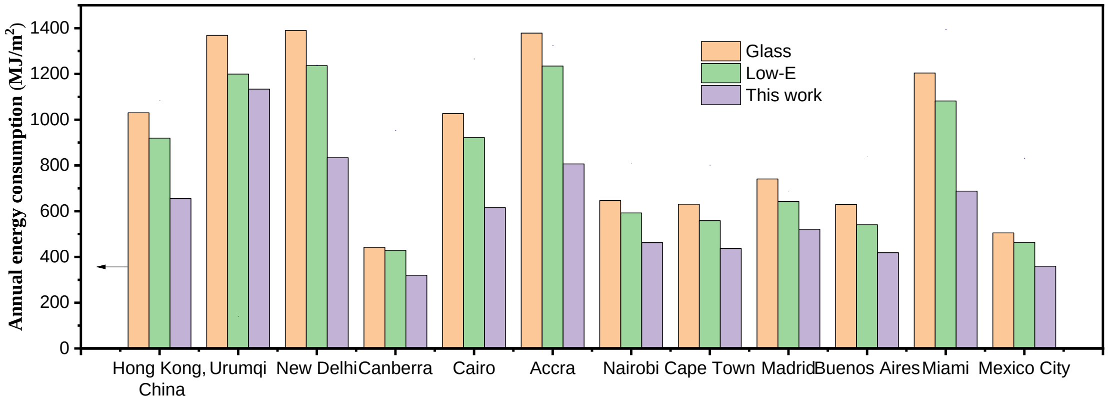

Fig. S9. AEC of the MTH based smart window house, the glass window house and Low-E window house are calculated for the globally typical cities.

Fig. S10.

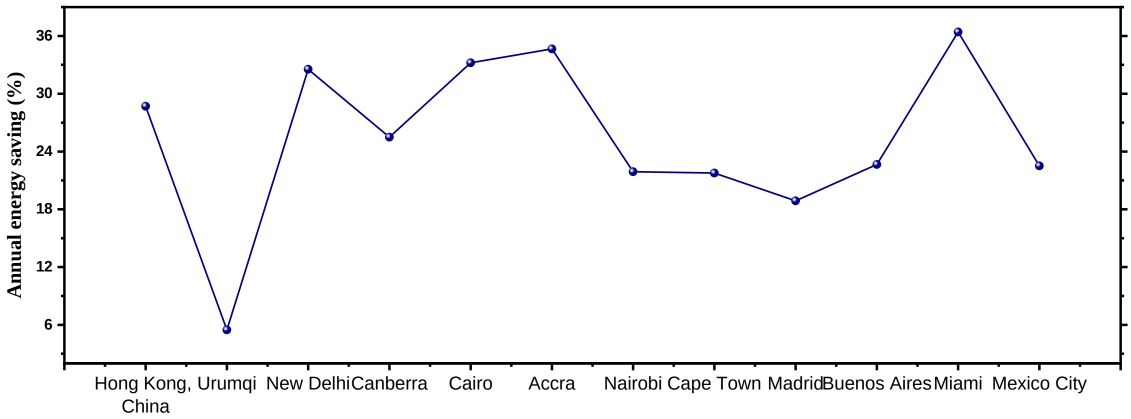

Fig. S10. The energy saving ratio of MTH based smart window house compared with Low-E window house

Fig. S11.

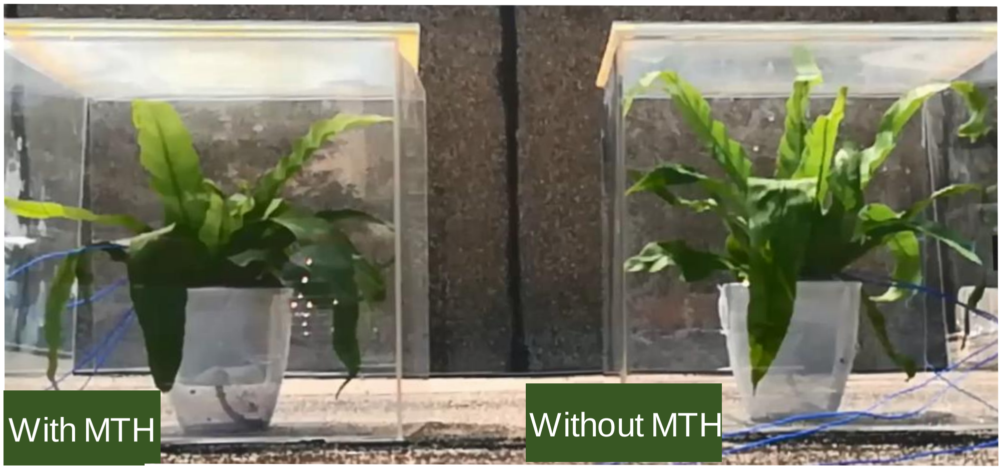

Fig. S11. The photo for the outdoor experiments of using MTH in smart plant factory. This picture demonstrates the experiment device for plant with/without MTH under the direct sunlight exposure.

Fig. S12.

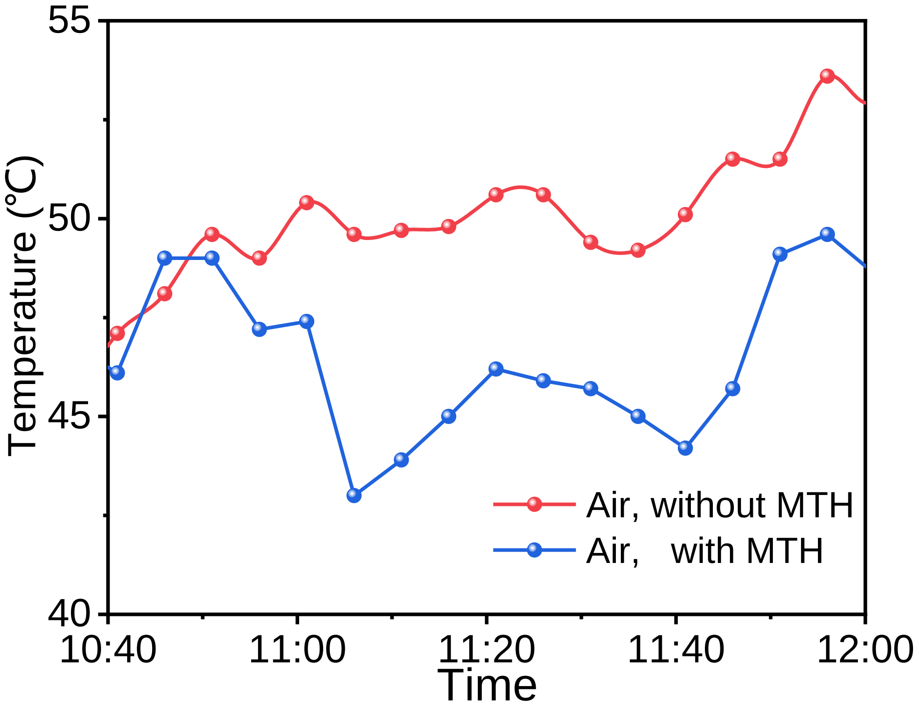

Fig. S12. The curves of inside air temperature of plants with/without MTH

Fig. S13.

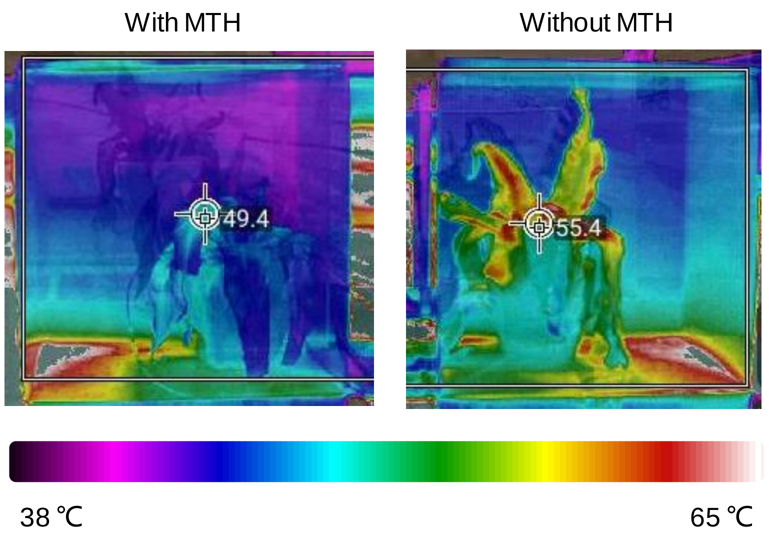

Fig. S13. Infrared images of the plants with/without MTH

Fig. S14.

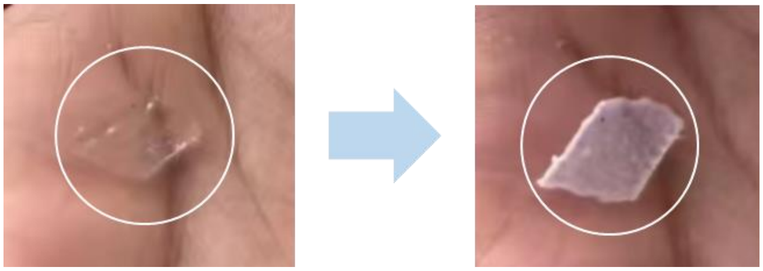

Fig. S14. The transparency changing state of anti-counterfeiting MTH touched by hands within 2 minutes.

Fig. S15.

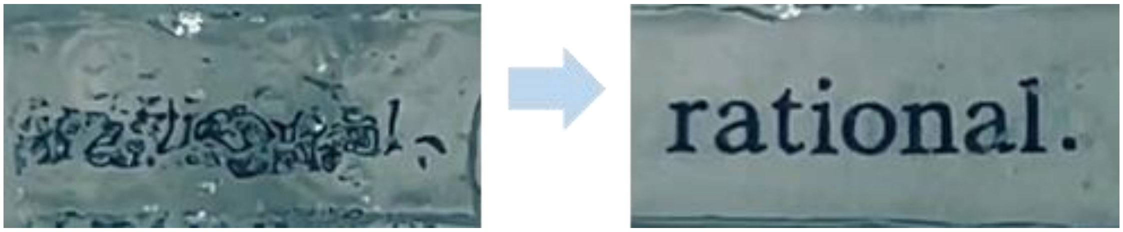

Fig. S15. The intelligibility changing state of anti-counterfeiting MTH pressed by pressure.

Fig. S16.

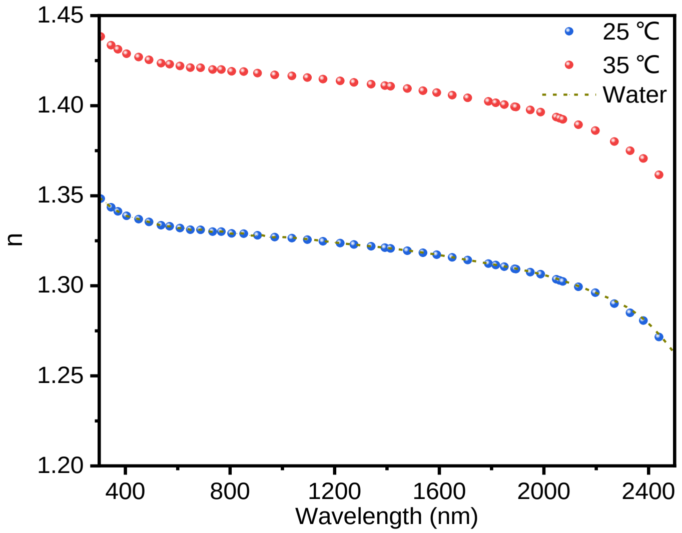

Fig. S16 The refractive index of the hydrogel adapted obtaining from Ref [17]

Table S1 Information of the building model applied in energy simulation.

Items	Specifications
Window fraction (window-to-wall ratio)	33.3% of above-grade gross walls
Window locations	Even distribution among all four sides
Glazing sill height	0.5 m
Exterior walls	Stucco + concrete + wall insulation + gypsum
Roof	Roof membrane + roof insulation + metal decking
Interior design temperatures	Winter: 18 ℃<br>Summer: 26 ℃

Table S2 Optical properties for different windows used in simulation.

Optical properties	MTH		Glass*	Low-E glass*
	Transparent	Opaque		
Solar transmittance	77%	15.6%	85%	63.0%
Visible transmittance	87.1%	16.9%	90.1%	85.0%
Emissivity (outer)	90%	90%	84%	84%
Emissivity (inner)	90%	90%	84%	10%

* Energy plus

Legends for Movies S1 to S4

Movie S1 (separate file). The MTH is applied to visible stealth of military tank. Under emergency conditions, the military tank model is invisible transiently after releasing the pressure stimulus.

Movie S2 (separate file). The MTH is applied to plant protection. The plant leaves turned from green to yellow after 20 min of sunshine without the MTH, while plant leaves remain green after 20 min of sunshine with the MTH.

Movie S3 (separate file). The MTH is applied to dual-level anti-counterfeiting. When the MTH is touched by hands within 2 minutes, the MTH becomes from transparency to opaque.

Movie S4 (separate file). The MTH is applied to dual-level anti-counterfeiting. the MTH shows characters below after pressed by a glass cover.

Reference

50. N. Garcia, E. Stoll, Monte Carlo calculation for electromagnetic-wave scattering from random rough surfaces. Physical Review Letters, 52(20) (1984) 1798-1801.

51. Tang K, Dimenna R A, Buckius R O. Regions of validity of the geometric optics approximation for angular scattering from very rough surfaces. International Journal of Heat and Mass Transfer, 1996, 40(1): 49-59.

52. Chen L, et al. Investigation of a novel frosted glass with regular pit array texture. Journal of Materials Processing Technology, 2016, 238: 195-201.

53. Bartell F O, Dereniak E L, Wolfe W L. The theory and measurement of bidirectional reflectance distribution function (BRDF) and bidirectional transmittance distribution function (BTDF)//Radiation scattering in optical systems. SPIE, 1981, 257: 154-160.

54. US Department of Energy, "EnergyPlus Weather Data" (2023); https://energyplus.net/weather.

55. Zhang Z, et al. Thermal conductivity of PNIPAm hydrogels and heat management as smart windows. Macromolecular Materials and Engineering 2023, 308, 2200566.

56. Tempel L V D, Melis G P, Brandsma T C. Thermal conductivity of a glass: I. Measurement by the glass–metal contact. Glass Physics and Chemistry 2000, 26(6), 606–611.
